# Extending cBioPortal for Therapy Recommendation Documentation in Molecular Tumor Boards: Development and Usability Study

**DOI:** 10.2196/50017

**Published:** 2023-12-11

**Authors:** Christopher Renner, Niklas Reimer, Jan Christoph, Hauke Busch, Patrick Metzger, Melanie Boerries, Arsenij Ustjanzew, Dominik Boehm, Philipp Unberath

**Affiliations:** 1 Chair of Medical Informatics Friedrich-Alexander University Erlangen-Nuremberg Erlangen Germany; 2 Group for Medical Systems Biology Lübeck Institute of Experimental Universität zu Lübeck Lübeck Germany; 3 Campus Lübeck University Cancer Center Schleswig-Holstein University Hospital Schleswig-Holstein Lübeck Germany; 4 Junior Research Group (Bio-) Medical Data Science Faculty of Medicine Martin-Luther-University Halle-Wittenberg Halle Germany; 5 Institute of Medical Bioinformatics and Systems Medicine University of Freiburg Faculty of Medicine University Medical Center Freiburg Freiburg Germany; 6 Partner Site Freiburg German Cancer Consortium (DKTK) German Cancer Research Center (DKFZ) Heidelberg Germany; 7 Institute of Medical Biostatistics, Epidemiology and Informatics (IMBEI) University Medical Center of the Johannes Gutenberg University Mainz Mainz Germany; 8 University Cancer Center University Medical Center of the Johannes Gutenberg-University Mainz Mainz Germany; 9 Medical Center for Information and Communication Technology Universitätsklinikum Erlangen Erlangen Germany

**Keywords:** molecular tumor board, documentation platform, usability evaluation, cBioPortal, precision medicine, genomics, health information interoperability, tumor, implementation, cancer, tool, platform, development, precision, use, user-centered

## Abstract

**Background:**

In molecular tumor boards (MTBs), patients with rare or advanced cancers are discussed by a multidisciplinary team of health care professionals. Software support for MTBs is lacking; in particular, tools for preparing and documenting MTB therapy recommendations need to be developed.

**Objective:**

We aimed to implement an extension to cBioPortal to provide a tool for the documentation of therapy recommendations from MTB sessions in a secure and standardized manner. The developed extension should be embedded in the patient view of cBioPortal to enable easy documentation during MTB sessions. The resulting architecture for storing therapy recommendations should be integrable into various hospital information systems.

**Methods:**

On the basis of a requirements analysis and technology analysis for authentication techniques, a prototype was developed and iteratively refined through a user-centered development process. In conclusion, the tool was evaluated via a usability evaluation, including interviews, structured questionnaires, and the System Usability Scale.

**Results:**

The patient view of cBioPortal was extended with a new tab that enables users to document MTB sessions and therapy recommendations. The role-based access control was expanded to allow for a finer distinction among the rights to view, edit, and delete data. The usability evaluation showed overall good usability and a System Usability Scale score of 83.57.

**Conclusions:**

This study demonstrates how cBioPortal can be extended to not only visualize MTB patient data but also be used as a documentation platform for therapy recommendations.

## Introduction

### Background

In molecular tumor boards (MTBs), clinical and molecular genetic data from patients with cancer, with a focus on those who lack standard treatment options or have rare tumors, are analyzed and discussed by oncologists, pathologists, and bioinformaticians regarding similarities, abnormalities, and possible new findings. The main goal is both to use advanced molecular genetic diagnostics and clinical assessment to provide therapy recommendations and to gather new insights and potential indications for highly personalized and genome-based therapy recommendations. Such MTBs combine research with patient care and are thus increasingly being implemented by oncologists around the world, as initial studies have shown a benefit for overall patient survival [1-3].

Despite the promising opportunities offered by MTBs, they still face various challenges that need to be addressed. These include the structured documentation of molecular data alongside clinical data and the resulting therapy recommendation in accordance with internal hospital guidelines and patient protection guidelines [4]. However, these data protection guidelines are yet to be uniformly designed and thus handled differently in each hospital [5], requiring local implementation of interfaces to patient records and laboratory systems. Although the transition from paper-based solutions to structured tools such as the electronic health record (EHR) is well advanced, patient-specific MTB therapy recommendations are still often designed as unstructured, free-text fields [5]. Hospitals use either self-programmed software solutions or a combination of various text editors and prefabricated forms, as shown by Hinderer et al [5] in 5 German hospitals. In these hospitals, the genomic data are recorded and communicated electronically or via paper, but in all cases, they are documented as free text without a coordinated data structure. However, this unstructured and nonstandardized state leads to the poor traceability of treatment evidence and decisions, making retrospective or follow-up studies, data sharing, and research projects difficult or even impossible.

Therefore, it is of utmost importance to document MTB recommendations and decisions based on molecular diagnostics in a structured and digital manner to standardize therapy recommendations and medical outcomes across clinics and sites with uniform data formats and reporting rules with the aim to improve patient care, as implied by Buechner et al [6].

Within the Medical Informatics in Research and Care in University Medicine (MIRACUM) consortium [7], the open-source platform cBioPortal [8,9], originally designed as a research platform for storing, analyzing, and visualizing omics data, is used to support MTBs.

To reach this goal, the platform is being adapted, extended, and integrated into university hospital networks so that data can be exchanged among the EHR, the laboratory systems, and the extended cBioPortal [10]. To promote structured therapy recommendations, we developed a therapy recommendation documentation module in cBioPortal, which is suitable for web-based use during MTB sessions as well as for preparation for upcoming MTB sessions.

Therapy recommendations are stored using Health Level Seven Fast Healthcare Interoperability Resources (FHIR) in compliance with the implementation guide “Genomics Reporting” and its German counterpart “Molekulargenetischer Befundbericht” by an FHIR-capable service running alongside cBioPortal [11]. This enables interoperability with the hospital information system (HIS). The standard cBioPortal data model is used for the storage of clinical and molecular patient data. During the development of the module, in addition to developing a standardized way to input the data, the focus was particularly on assuring high usability of the interface for the actual users, as this is a crucial point for the acceptance of a new tool and can even impact the operator’s therapeutic decisions, as shown by Bates et al [12]. To enable secure and compliant use of the data in the hospital network, IT security was taken into account from the beginning of the project. This means that the development was based on best practices regarding the chosen protocols and used interfaces. However, the developments in the area of IT security mainly focus on enabling a connection among the newly created user interface, the HIS, and external backends to the authentication mechanism already existing in the open-source platform and introducing role-based access rights to the backends compatible with commonly used identity providers (IdP). Other security-related aspects, such as enforcing encrypted communication in the network, identifying existing attack vectors, and following all legal and organizational guidelines, must be addressed by the responsible parties during integration into the target network of the respective partner sites.

### Objectives

The cBioPortal research platform was expanded with a module that (1) is linked to EHRs and HISs, facilitates the digital documentation of therapy recommendations in MTB sessions in a structured manner, and (2) enables digital accessibility for all persons and partner sites involved in research projects. This allows the standardization of documentation processes across partner sites and supports subsequent collaboration between research and patient care. Therefore, the extension had to be compliant with IT security standards to protect patient data. Furthermore, the solution should be largely independent of the specifics of the HIS in use by providing portable interfaces, which ensures that it can be put into operation at additional sites without major migration effort and thus contribute to an expansion of the research network.

The first objective was to demonstrate the extent to which the solution can harmonize the currently used documentation processes for MTBs across sites. The second objective was to engage future users in a user-centered design approach by applying an iterative feedback process [13-15] to ensure the quality of the module’s use and user satisfaction during the MTB workflow. Finally, to further involve users in the development process and to test the success of this approach, a usability evaluation of the module’s web interface was conducted.

## Methods

### User-Centered Design Approach

During the development of the application, a user-centered design approach was used, which consisted of a requirements analysis together with an iterative feedback process including all partner sites, to improve the usability and, thereby, the clinical applicability of the resulting tool. A total of 12 experts from 6 of the 10 partner sites were involved in this process. They covered a wide variety of backgrounds, including oncology, systems medicine, bioinformatics, and IT, and all of them belonged to the circle of future users. Although the exact processes and roles of an MTB vary from site to site, the use scenario of the documentation platform can be roughly divided into 2 roles. First, there is a preprocessing and postprocessing team, which takes care of data maintenance and documentation. Second, there is a specialist team, which looks at the data and discusses the cases in the MTB. However, both roles can sometimes be performed by the same people, depending on the responsibilities in the site. It was ensured that people from both roles were involved in the user-centered design approach. The selection of additional technologies and frameworks required for authentication and ensuring IT security was elaborated by means of a technology analysis and compared with widespread standards and best practices.

In the first step, the experts specified the required data elements and types from a medical perspective. Subsequently, this information was converted into a data structure applicable to therapy recommendations to be used in the prototype implementation for a high-fidelity mock-up of the documentation module. The resulting mock-up was then iteratively refined in 3 feedback sessions with the help of future users. Each session consisted of a short evaluation with the users, in which they reported feedback or problems via unstructured free text. This feedback was converted into change requests, documented in a quality management system, and integrated into the mock-up before the next feedback session commenced.

### Prototype

After the completion of the extended user interface, the module was integrated into the front-end codebase of cBioPortal as a new tab in the patient view. For persisting the entered data, it was connected via a representational state transfer (REST) interface to a FhirSpark server that was used as a tightly coupled, secondary backend [11], resulting in the first fully functional prototype of the module. During the development of the front-end application as well as the backend connections and integrations, an agile approach was used, which was largely based on the adaptive software development concept [16]. The state of the prototype was extended in iterative steps, with the developers meeting at regular intervals with the project team and domain experts to evaluate the recent implementations and discuss the priorities and next features to be implemented from the design phase.

To provide a standardized and simplified way to install the whole setup, cBioPortal and the corresponding services were packaged as Docker containers [17] to make the solution independent of the operating system and use the easy deployment and distribution process of these containers. In the first step, the deployment workflow was tested at the University Hospital Erlangen, including the integration of the components with the HIS. In the second step, the tool was distributed to all 9 consortium sites of MIRACUM and 2 external partner sites to demonstrate that connection to the various systems in the hospital network can be established without major effort and is almost independent of the specific HIS in use. This test was accompanied by structured feedback questionnaires to evaluate whether the general installation was successful, the test data could be imported, and the various tools and annotation services were working as expected. The questionnaire was sent to the partner sites together with the documentation and was meant to be completed by the person responsible for setting up the system together with a key user of the application. This feedback was intended to show whether the planned goals for integrating external sites and distributing the modules can be achieved [10].

### Usability Evaluation

Finally, a usability evaluation was conducted using the MIRACUM-cBioPortal (version 2021q1 [18]). This evaluation aimed less at evaluating the resulting application in comparison with other existing tools and more at evaluating the application’s general usability in contrast to the previously unstructured approach, as a sufficient level of usability, which leads to users’ acceptance, is the basis for a later successful adoption into the clinical workflow. The evaluation was set up as a combination of a task analysis with the thinking-aloud technique, as this approach focuses on the user needs and behaviors while they perform the tasks and can facilitate and accelerate further usability enhancements. In addition to the qualitative evaluation, several quantifiable metrics regarding the time and effort users spent on interacting with the system while completing the tasks were established to determine the extent to which the resulting web interface could meet the self-imposed requirements for improving the documentation process. During the test, users were guided through a series of 8 specific tasks designed to evaluate the system’s usability and functionality while articulating their intentions and thoughts as they interacted with the web interface. These tasks encompassed actions such as logging in, creating a new MTB entry for a test patient, manually and automatically generating therapy recommendations, adjusting therapy priorities, and securely logging out. The tasks were formulated to closely mimic a typical workflow and test the essential features of the MTB software system. The users’ performance on each task, that is, the time it took them to complete the task and the number of errors they made, as well as their verbally articulated insights, was derived into metrics that allow conclusions about the difficulty of the task itself as well as the amount of time the users spent physically interacting and cognitively engaging with the web interface while working on the given task. After the tasks were completed by the prospective users, their perceived acceptance of and satisfaction with the module and workflow were measured by interviewing them using a structured questionnaire and the widely used System Usability Scale (SUS) [19] to obtain qualitative feedback as well. In these questionnaires, the users were asked about their level of agreement with preformulated statements about the platform (Multimedia Appendix 1) and performed tasks using a 5-level Likert scale, which ranged from “strongly agree” to “strongly disagree,” allowing the derivation of an average satisfaction rate. Finally, the users were given the opportunity to share their opinions and additional subjective remarks to obtain further feedback about their acceptance of the developed module. This feedback was used to assess possible areas of improvement in the interface that could further increase the perceived usability.

### Ethical Considerations

The project was performed in compliance with the World Medical Association Declaration of Helsinki on Ethical Principles for Medical Research Involving Human Subjects. Ethical approval was not required.

## Results

### User-Centered Design Approach Results

**Figure 1:**
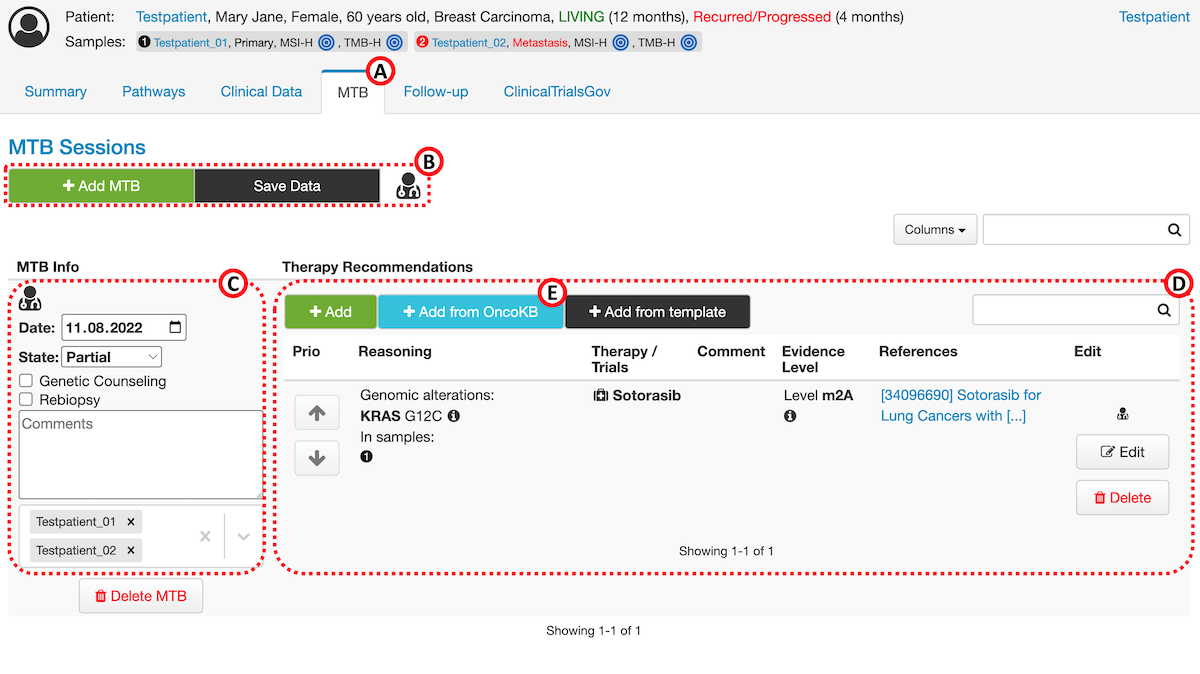
Overview of the extended cBioPortal patient view. (A) Newly created tab “MTB” for documenting therapy recommendations. (B) User interface for adding molecular tumor boards (MTBs), saving data, and user management. (C) General information of the MTB session. (D) Detailed documentation of the recommended therapies or trials. (E) Button for adopting matching OncoKB entries as therapy recommendations.

The harmonized data structure of the documentation module in the front end was developed using the iterative approach described earlier. The results showed that in addition to the general and organizational information about the MTB session and the medical data of the discussed patient, further therapy-related characteristics are required. Therefore, the data should include information such as the active substance or substances recommended for therapy, genetic alteration or alterations, complex biomarkers, and clinical data on which the recommendation is based, as well as the level of evidence (defined by the Zentren für Personalisierte Medizin). The clinical and genomic data of the respective patient are already stored in cBioPortal, whereas data about the active substances recommended and level of evidence had to be added and be selectable via a drop-down menu to avoid typing errors and inconsistencies during entry. To substantiate the evidence level, case reports and medical articles listed in the PubMed database [20] can be indicated by their ID, and a field for comments should be available for entering free text. In addition, the knowledge database OncoKB [21], which contains treatment implications of cancer gene alterations, was integrated. A separate button can be used to search for predefined treatment entries in this database and adopt them directly (Figure 1). The mapping of the entries derived from the external database into the module’s corresponding input fields is automated, and only the evidence level must be defined manually owing to the use of different evaluation scales. Finally, the design of the web interface was defined, as shown in Figure 1.

### Authentication Enhancement Results

To integrate the module into the HIS and, thereby, comply with the applicable regulations and guidelines for the later planned productive operation, further measures for authentication and authorization had to be implemented in addition to the security features already provided by cBioPortal. Unauthorized access to the data stored in the secondary backend had to be prevented, and a finer distinction between read and write permissions for the data sets had to be enabled.

By default, it is already possible to connect cBioPortal to an external IdP using the Security Assertion Markup Language (SAML) framework [22] to establish a secure connection between the systems and enable individual user log-in sessions. In this process, users can be authorized individually via the IdP for each of the imported studies. For this purpose, the widely used open-source software Keycloak (JBoss) [23] is used, which is supported by Red Hat (Red Hat, Inc) and was already in use at the hospital’s network. Keycloak is connected to the corresponding user databases via Lightweight Directory Access Protocol [24], whereby user information can be automatically imported to Keycloak and users can be granted access to cBioPortal, depending on their preassigned roles. However, the therapy documentation module requires the possibility of individually restricting access to patient data as well as a further differentiation of the rights for accessing, editing, and deleting the data. Therefore, it was necessary to set up an additional way of role-based access control for protecting the entered data and to generally restrict access to the FhirSpark backend server. For this purpose, with the lua-resty-openidc module [25], another open-source software was chosen. This module protects the backend by running directly on the nginx proxy server (Nginx, Inc) and can be configured to act as a relying party within the OpenID-Connect [26] authentication layer. For each incoming request, it verifies whether the caller is authenticated by Keycloak and, if so, forwards the user roles attached to the request so that the backend application server can process or deny the request according to the defined logic. Although this architecture uses 2 different protocols, the users are not negatively affected because they only have to log in once via Keycloak. When accessing individual resources, the IdP automatically takes care of the log-in and user role forwarding in the background via single sign-on (SSO). The advantage of outsourcing the access restriction to the nginx proxy server is that with this setup, additional backends can easily be integrated into the SSO realm without them having to deal with authentication protocols, as this is already taken care of by the proxy server.

### Prototype Results

**Figure 2:**
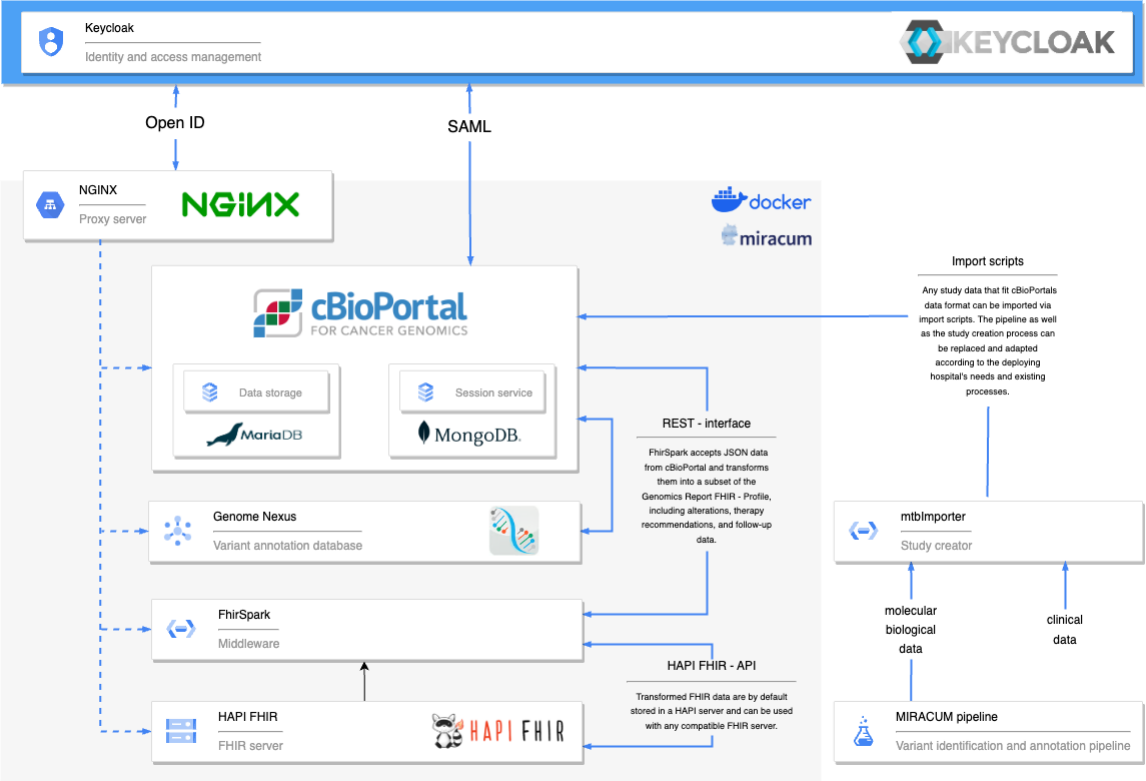
Detailed system overview of the extended cBioPortal and the associated services that together form the software platform for supporting molecular tumor boards. API: application programming interface; FHIR: Fast Healthcare Interoperability Resources; HAPI: HL7 Application Programming Interface; MIRACUM: Medical Informatics in Research and Care in University Medicine; REST: representational state transfer; SAML: Security Assertion Markup Language.

For the evaluation of the tool across partner sites, the dockerized workflow was made available via GitHub (GitHub, Inc) [18]. This project combines the extended cBioPortal front end and backend as well as the FhirSpark server, additional annotation services, and further modules for authenticating and authorizing users through a preconfigured nginx proxy server. A detailed system overview is depicted in Figure 2. The tool was deployed and evaluated at 11 partner sites, including the integration of local authentication and authorization services. The study by Reimer et al [10] provides a detailed overview of the evaluation results. Configuring and testing the authentication setup and the subsequent transmission of therapy recommendations to the FhirSpark backend were successful at 4 (36%) of the 11 sites. At the remaining sites (n=7; 64%), the setup was not tested because either a decision was made not to use an authentication service or configuration problems had already occurred at an earlier step, because of which a connection to the secondary backend could not be established. Feedback suggested that the documentation and setup instructions should be extended and adapted to the errors that arose. For example, in addition to listing the minimum hardware requirements and the recommended software versions to rule out errors due to insufficient server configuration, the documentation should be expanded to include an even more detailed description of the Keycloak integration.

### Usability Evaluation Results

Among the 12 experts who participated in the user-centered design approach, 7 (58%), all of whom were working in the fields of molecular biology, oncology, and systems medicine and belonging to the group of potential future users, participated in the subsequent usability evaluation conducted to review the extended front-end module. The evaluation showed that, on average, the test users were 89% (SD 19.7%) confident that the enhanced platform can support them with their specific documentation tasks and 71% (SD 36.6%) confident that it can also support the general approach to conducting MTB sessions. With regard to the usability of the web interface itself and the input dialogs, an average satisfaction of 75% (SD 25%) was indicated in terms of the perceived comprehensibility and clarity of the user interface. In addition, the authentication features in particular were rated as satisfactory, with 82% (SD 18.9%) satisfaction in terms of user-friendliness, meaning that the users predominantly did not perceive authentication as disrupting their workflow. The mean score on the administered SUS was 83.6 (SD 14.3). On the basis of the results of the qualitative questionnaire, there is general comprehensibility of the web interface, although in some cases, the correct assignment of clinical data to the designated input fields was rated as not clear enough. For example, some participants attempted to enter relevant biomarkers such as the tumor mutational burden (TMB) value directly into the field for single genomic alterations, possibly thinking that the TMB should be directly assigned to the respective alteration. However, a separate field is provided for this purpose, as values such as the TMB refer not to an alteration but to a specific sample. Apart from providing minor assistance with entering free text so that it gets automatically applied when typing, allowing formatted comments, and more intuitive labeling of the log-in button, no major changes to the user interface were requested. Furthermore, the test users recommended that actual users be given a brief introduction to all the available functionalities at the beginning of productive operation.

**Figure 3:**
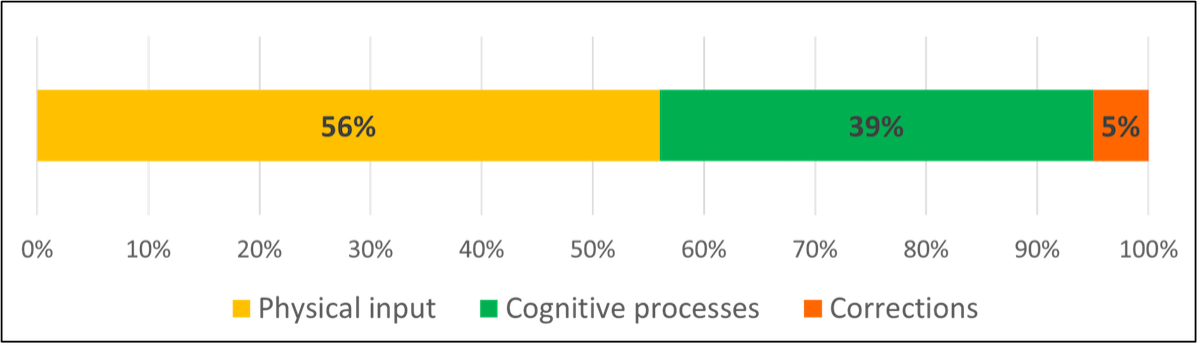
Temporal distribution of the user interaction processes.

**Figure 4:**
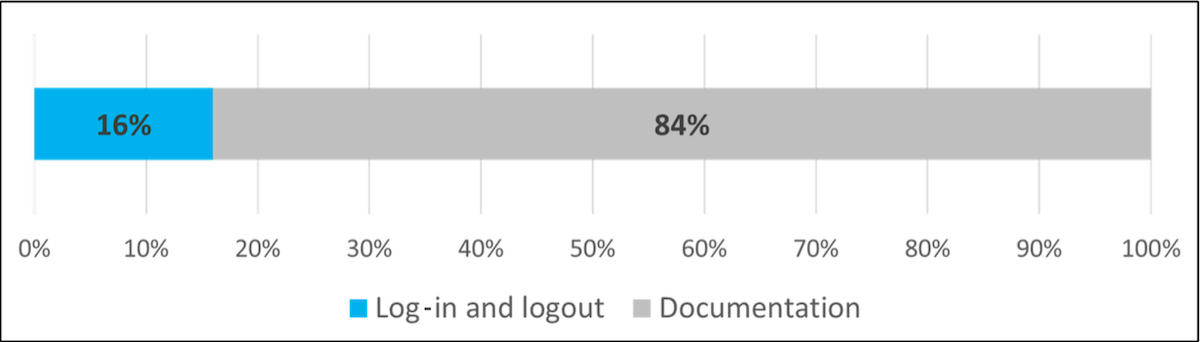
Temporal distribution of the authentication process.

For the quantitative evaluation of the task analysis, the proportions of time spent thinking and idling, with thinking and idling combined as cognitive processes, and time spent physically interacting with the web interface while executing the tasks in relation to the total time needed as well as the proportions of time spent for log-in and logout activities were collected. The results are depicted in Figures 3 and 4 and reveal that the actual physical tasks of interacting with the interface and entering new data accounted for the largest share of time spent at 56% (mean 190, SD 70 seconds) of the total time needed, followed by the cognitive processes at 39% (mean 267, SD 101 seconds). A total of 5% of the time was spent making corrections to previously entered data. The process of logging in and out of the platform, as well as verifying the authentication status, accounted for an average of 16% of the total interaction time.

## Discussion

### User-Centered Design Approach Discussion

A new therapy documentation module for structured therapy recommendations in cBioPortal has been developed using a user-centered design approach and was tested with regard to its functionality and usability. As this was only a prototype implementation, there is still potential for additional enhancements and improvements in usability. The iterative development process revealed its importance through the users’ identification of additional requirements at different stages of the prototype. It was found that maximizing the number of feedback rounds with the users provided valuable insights, although the frequency of these rounds was constrained by user availability and the development timeline. Comprehensive documentation was emphasized as crucial for facilitating the deployment phase, allowing for easier adoption and integration of the tool into existing workflows.

### Authentication Discussion

To ensure compliance with information security goals, with the SAML framework, a standardized authentication technology is used to protect the connection between the software and the IdP of the hospital. cBioPortal already supports SAML by default; however, no practicable way could be identified to use SAML with the externally developed FhirSpark server, so OpenID-Connect was chosen instead as the security technology for connections to this application. As both protocols are widely used and most IdPs support them, it is possible to flexibly switch from Keycloak to another IdP while maintaining the existing setup and configuration without the need for major adjustments. The use of a proxy server in front of the different backends that takes care of the authentication protocols and forwards the user roles promotes subsequent adaptability to different network environments and the possibility to easily connect additional applications to the same role-based access control system. Furthermore, it is possible within the IdP to enable an SSO session between both protocols and across the several connected backends. With this SSO session, the IdP takes care of the authentication and authorization flow in such a way that users need to log in only once, but their user session is automatically authenticated at all the backends connected via the protocols without losing the advantages and flexibility that come from having 2 security mechanisms in place, with each one suited for its specific purpose. Evidently, certain applications can be excluded from the SSO, and the user permissions can be revoked as necessary. Another possible expansion of the functional scope and, at the same time, a broader differentiation of user permissions for the therapy recommendation module would be the introduction of explicit rights for the deletion of data, which would split up the previously existing editing rights into 2 separate scopes. This would enable the realization of further distinction possibilities between these operations, as there may be use cases where a user has the permission to create or edit entries but not to delete an entire MTB session for traceability and other security reasons. This possible introduction of different rights for the editing and deletion of data was specifically requested by the users; however, in the backend server, there are logging mechanisms that enable the traceability of data records and user actions.

### Prototype Feedback

The decision to use a secondary backend for data persistence in the prototype implementation instead of using the existing internal cBioPortal database is based on the fact that the cBioPortal database is primarily intended as a repository with read-only access to patient data without frequent changes. By contrast, the FhirSpark module is specially designed for changing data sets and provides support for traceability and version control. Furthermore, with each new release of the official cBioPortal backend, the differing database structures would have to be reviewed and potentially merged, which would require an additional maintenance effort. In addition, the proprietary backend offers a higher potential for interface customization and access control, allowing additional external applications to be connected to the database as needed with little effort.

By distributing the prototype implementation to several locations and receiving feedback on the installation and first practical tests, it was determined that the distribution of the application as Docker containers is a suitable method for deploying the software at all partner sites almost independently of the operating systems used and, thereby, accelerating the installation process [10]. This also facilitates the subsequent updates of new versions and changes to individual components. The successful setup and deployment of the platform’s basic functionalities were confirmed by all sites, and the newly added components for IT security were successfully connected to the existing solutions and tested at several sites. However, at some sites, problems were encountered at various points in the configuration process, indicating that the documentation needs to be more detailed to cover all the edge cases and specifics of the different IT infrastructures used. To reduce the complexity of the installation process while testing, integrating the tool into the HIS with authentication services was highly recommended but only optional. Although this led to a significant adoption of the tool in general, little feedback was obtained on additional features such as authentication and connection to other systems. Some sites have not implemented these features because of time constraints; others used systems different from Keycloak for identity management, for which configuration instructions were not provided, although integration would be possible. Overall, the feedback provided several indications on how to improve the documentation for future releases as well as further suggestions for minor usability improvements to the user interfaces.

### Qualitative Usability Evaluation

Regarding the functionalities offered by the enhanced web interface and the user satisfaction achieved while documenting the therapy recommendation, both indicators were rated as fundamentally positive and satisfactory by the users who participated in the usability evaluation. This shows that the future users are convinced that the module has the potential to digitalize the current processes and documentation data in a structured form and fulfills the general requirements for supporting the existing documentation processes and the expectations of the users. The somewhat lower assessment of the perceived overall support potential for MTB meetings (71%), in contrast to the assessment of the individual functionalities, can be explained by the fact that many sites already use locally established documentation processes, such as their own proprietary implementations or prefabricated text-based document templates. Therefore, converting all workflows to the integrated infrastructure is considered too costly and accepted by stakeholders only with reservations. Another issue is that some users mistakenly related the purpose of the evaluation not only to documentation tasks but also to the whole process of an MTB, starting with case preparation and data analysis. Therefore, the questions were answered from different perspectives. Nevertheless, further feedback should be sought on how to further increase the perceived potential among users. Altogether, the achieved SUS score of 83.6 is above average and can be rated as “excellent” [27] or graded as “A” [28], depending on the evaluation standard applied. Thus, the SUS confirms the results of the structured feedback questionnaire on user satisfaction with and support in using the module. It should be mentioned that the concrete SUS score should not be overinterpreted and can only show a tendency.

### Quantitative Usability Evaluation

As the temporal distribution of the processes shows, the users spent most of their time physically entering the data. This is expected because the module is primarily a documentation tool. On the contrary, a predominant share of mental processes would be an alarm sign that the structure of the interface is too complicated. At 39%, the mental processes are nevertheless at a relatively high level, which is due to the fact that most users were using the platform for the first time ever at the time of the evaluation and, therefore, had to first familiarize themselves with the design of the site. In addition, the molecular biology background plays an important role, as the validity of the entries must also be checked during the documentation process and compared with the patient data to avoid errors. The amount of time required to complete the authentication tasks is relatively high (16%). However, this can be explained by the fact that, as part of the tasks, the users had to explicitly check whether the log-in and logout processes were successful and whether they had been granted the correct user rights. After logging out, they also had to actively check whether the therapy recommendations could no longer be modified as part of the test. In the routine workflow, these steps are omitted, and the log-in process needs to be performed only once per session. This will significantly reduce the time spent on authentication tasks. This is also supported by the qualitative feedback from the test users, as the authentication function was subjectively rated as not hindering the workflow and overall perceived as positive.

Furthermore, it can be stated that the participants basically coped well with the operation of the platform but that a short introduction to all the functionalities, user dialogs, and submenus and further test runs before the integration of the platform into the daily clinic routine are considered helpful for quick familiarization. Major changes to the design are not necessary, but the minor suggestions for improving usability that were most frequently mentioned in the feedback, such as the instant adoption of typed text and the sorting of the entered MTB sessions, should be addressed to further improve the quality of use.

### Comparison With Other Systems

Although several projects aim to provide an MTB platform and focus on different use cases, none of them yet provide a standardized means of documenting MTB recommendations and decisions. The Molecular Tumor Board Portal of Tamborero et al [29] offers a clinical decision support platform to analyze Variant Call Format files and generate HTML reports with annotated and interpreted variants. An internal and further expanded version is used by 7 comprehensive cancer centers to analyze sequencing data harmonized with respect to data privacy, state of research, and technological implementation. The platform can be used for joint case discussions, but the documentation thereof is not supported by the portal.

The “VITU – Virtuelles Tumorboard” tool [30] aims to support and digitize MTBs by serving as an information and communication platform. In addition to facilitating MTBs with a videoconferencing tool integrated into the platform and a digitized, process-based case review option, further solutions for integrating external experts will be offered, according to their website. It also contains a module for structured documentation in combination with an FHIR interface for accessing the data. Although there is a demo version of the software available for web-based testing and the source code is available on GitHub, it is not yet conceivable how the software would perform in productive operations, as it still seems to be in the development phase, and updates seem to have slowed down since version 2019.3 [31].

A similar purpose in terms of supporting and promoting MTBs is pursued by Alteration Annotations for Molecular Tumor Boards [32]. This is an R shiny–based web application developed by researchers at the University Hospital in Ulm and consists of multiple modules that, in addition to the visualization and annotation of mutations, offer the possibility of displaying the evidence for possible therapeutic drug targets. Thereby, the identified mutations can be evaluated, discussed, and processed in various formats so that the findings can be exported to external clinical systems. Therefore, the public databases GDKD, CIViC, and TARGET [33-35] can be connected, which are used as a knowledge basis for variant annotations by linking together various information sources. Overall, the application, which is based on the R programming language, is primarily designed for the preparation and visualization of data but also includes functions for highly automated data integration and the standardized documentation of results. However, the capability to share data across sites, specifically for research purposes, appears to be limited.

Schapranow et al [36] have created an advanced software tool designed specifically for multidisciplinary tumor boards. Their approach involved a thorough requirements analysis and followed a user-centered development process. The software allows a dedicated person to note down therapy recommendations, clinical trials, and additional remarks live and transparently on a presentation screen visible to the attendees. The software then automatically generates a report upon the completion of the structured documentation, which is provided to the treating physician. In addition, the software integrates external data sources in a modular manner, focusing on the specific requirements of multidisciplinary tumor boards, and builds the technical foundation of a multisite database of participating hospitals. Although the software is still in the prototype phase, its modular design should enable easy integration with existing clinical documentation.

Comparing the functional scope of cBioPortal in combination with the newly created module with that of similar, already existing systems, it becomes apparent that the integration of a standardized documentation tool for the cross-site harmonization of findings and therapy approaches can create an added value that is not yet fully exploited by other solutions. The possibility of integrating the tool into the clinic’s internal workflow and additionally connecting other external systems and data sources to it holds great potential, particularly with regard to the exchange of research data for the transfer of knowledge and the promotion of innovation between clinic alliances and research networks that this makes possible. Thus, there are many more use cases that can be addressed beyond just data visualization and storage, such as fostering collaboration between research and patient care and facilitating the selection and inclusion of appropriate data sets and patients for studies and research projects. That this is a contemporary application field with great potential is demonstrated by the National Network of Genomic Medicine, in which personalized therapy for patients with lung cancer is supported through the provision of a common database and facilitated by the use of a central study registry [37].

Finally, the question of whether the developed solution might be used for similar applications, especially organ tumor boards, arises. Although there is a significant overlap in the general requirements, as well as some overlap in the underlying data model, cBioPortal is primarily tailored to molecular biological data and their visualization. Therefore, it is not an adequate software basis for other tumor board applications without significant alterations. That said, the general approach to design and development is replicable and may be adapted to develop solutions for related problems. One of the key points of this study that can be generalized to the field of medical informatics is that it is crucial to develop tools that adhere to established standards, especially using FHIR and harmonized implementation guides. This ensures seamless integration. In addition, consistently prioritizing user requirements is essential for effective and efficient support for health care delivery.

### Outlook

Using the feedback from the usability evaluation, the tool will be refined and adapted to provide users with a comprehensive documentation platform for MTBs. Subsequently, an additional tab will be developed to enable the documentation of follow-up data based on the recommended therapies to further expand the documentation capabilities of cBioPortal. This also paves the way for building a standardized and multisite pool of MTB data, which can later be used for the annotation of new patients and as input for machine learning techniques to generate new insights about MTB treatment and outcomes. An important aspect of extending the tool is compliance with the Medical Device Regulation and In Vitro Diagnostic Device Regulation. As the software currently supports (in the context of Medical Device Coordination Group 2019-11 [38]) only simple functionalities, such as storage, archiving, communication, and simple search, it does not fall within these regulations.

### Conclusions

In summary, a custom module for the cBioPortal platform was implemented to enhance the platform with functionalities for documenting therapy recommendations in a harmonized manner across collaborating hospitals and research sites, and an interface for enhanced authentication and authorization purposes was added to comply with security regulations and ensure the protection of patients’ data. The findings of the hands-on tests and the usability evaluation suggest that the resulting solution for the documentation of therapy recommendations can be deployed successfully at various sites independently of the HIS in use without much effort. The usability evaluation leads to the assumption that the module and user interface will be widely accepted by the future users and thus can be successfully integrated into their workflow. Therefore, the newly introduced functionalities have the potential to improve the existing documentation processes by providing a structured and harmonized digital template for documentation data. Thus, these functionalities could not only lead to a significant benefit by directly supporting the preparation and conduct of MTBs but also promote the development of further applications, leveraging the implemented harmonized data structure to reuse the collected data. Fields of possible applications are the postprocessing of MTBs and case preparation using the follow-up data of previous patients in future MTBs. In addition, opportunities for interdisciplinary exchange may arise through the transfer of innovation among different research groups to discover new medical relationships based on the accessibility and sharing of data among partner sites.

## Appendix

Multimedia Appendix 1 Statements of the quantitative questionnaire and items of the open feedback questionnaire (translated from German for publishing).

## Abbreviations

EHR: electronic health record
FHIR: Fast Healthcare Interoperability Resources
HIS: hospital information system
MIRACUM: Medical Informatics in Research and Care in University Medicine
MTB: molecular tumor board
REST: representational state transfer
SAML: Security Assertion Markup Language
SSO: single sign-on
SUS: System Usability Scale
TMB: tumor mutational burden
