# Supplementary material for: Extending cBioPortal for Therapy Recommendation Documentation in Molecular Tumor Boards: Development and Usability Study
Source: JMIR Med Inform. 2023 Dec 11;11:e50017. doi: 10.2196/50017 (PMC10750236; doi:10.2196/50017)
Supplement: Multimedia Appendix 1 [file medinform_v11i1e50017_app1.docx]

## Questionnaire Items

Statements of the quantitative questionnaire (translated from German for publishing):

1. The tasks set and the procedure were understandable.
2. The web interface and dialogs were always clearly arranged and understandable.
3. I was able to quickly find my way around the site.
4. The web interface assisted me well in completing the tasks.
5. The sign in / sign out did not take me away from the actual tasks.
6. I believe that the MTB tab can support me in my work at the Molecular Tumor Board.

Items of the open feedback questionnaire (translated from German for publishing):

1. Do you have any questions about the web interface that need clarification?
2. Did you encounter any issues with elements or their functionalities on the website?
3. Were you able to provide all the relevant data for therapy recommendations?
4. Would you have liked to provide or receive additional information about the patient or the case?
5. Did you encounter any issues during login or logout? (If yes, could you specify?)
6. What did you find particularly problematic that should be changed?
7. What did you find particularly effective that should be retained?
8. Please briefly describe your field of expertise and how you usually document therapy recommendations.
